# The challenge of reviewers scarcity in academic journals: payment as a viable solution

**DOI:** 10.31744/einstein_journal/2024ED1194

**Published:** 2024-09-09

**Authors:** José Belém de Oliveira

**Affiliations:** 1 Hospital Israelita Albert Einstein São Paulo SP Brazil Hospital Israelita Albert Einstein, São Paulo, SP, Brazil.; 2 Universidade de São Paulo São Paulo SP Brazil Universidade de São Paulo, São Paulo, SP, Brazil.

Editors-in-Chief of academic journals worldwide are encountering a shared challenge: the struggle to find scholars and researchers willing to dedicate time to reviewing research articles. Peer-reviewing, a cornerstone of academic publishing, often involves double-blind assessment by at least two anonymous experts from the scientific community before dissemination.^(1)^ Although peer-reviewing is a common practice in science, the growth of research output globally and the amount of time needed by reviewers to prepare their revisions seems to go in opposite directions.^(2)^

In recent years, the so-called ‘reviewer fatigue’ phenomenon has increased, being further exacerbated during the COVID-19 pandemic.^(3)^ A report by Publons in 2018^(2)^ on the Global State of Peer Review pointed out that, in 2017, Editors-in-Chief needed to send an average of 2.7 invitations for every accepted review. Projections suggest this average would rise to 3.6 invitations per acceptance by 2025.

In an attempt to solve the scarcity of reviewers and acknowledge their invaluable voluntary activity, some members of the scientific community have been suggesting payment of reviewers as a possible solution.^(4,5)^ The number of hours that reviewers often spend is surprising high. According to a study,^(6)^ during the year of 2020, reviewers worked over 130 million hours doing reviews. In the same year, the estimation is that US-based reviewers alone spent US$1.5 billion on reviewing.^(6)^

Despite the high numbers of hours and substantial amount of money, the concept of payment is controversial, and many scholars advocate that peer review should be a free service to the academic community. Those opposing payment believe that everyone publishing will end up needing to have their paper peer reviewed. Payment is also criticized by journals not affiliated with large publishing companies, which normally operate on limited budgets. Payment, however, is not limited to money; alternative forms of recognition have been proposed, including credits to offset articles processing charges, formal recognition from universities and organizations, and certificates.

In light of the sensitivity surrounding this topic, journals have promoted surveys to learn their reviewers’ opinion on the subject. Recently, einstein (São Paulo) journal, the official scientific publication of *Instituto Israelita de Ensino e Pesquisa Albert Einstein*, conducted a survey (between late 2022 and early 2023) aimed at understanding factors influencing reviewers’ decision to accept review invitations, opinions on rewarding reviewers, and preference for type of attractive compensation. Out of 1,000 reviewers surveyed, 329 academics, mostly from healthcare field, responded.

## To review or not to review?

**Figure 1:**
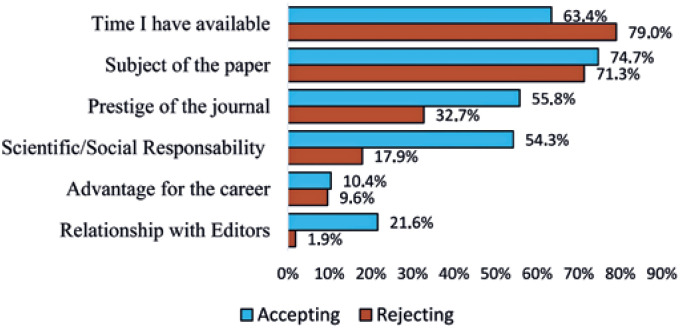
Factors impacting the decision of accepting or rejecting peer reviews

For the participants, factors that most affected their decision on accepting to review a paper was the *subject of the paper* followed by *time I have available for reviewing a paper*, *prestige of the journal*, *feeling of scientific/social responsibility,* *relationship with the associate editor* and, *clear advantage for the career*. Meanwhile, factors that count on their decisions the most for rejecting a review were *time I have available for reviewing a paper*, followed by *subject of the paper*, *[lack of]* *prestige of the journal*, *feeling of scientific/social responsibility, clear advantage for the career* and, *relationship with the associate editor* (Figure 1).

## To reward, or not to reward?

**Figure 2:**
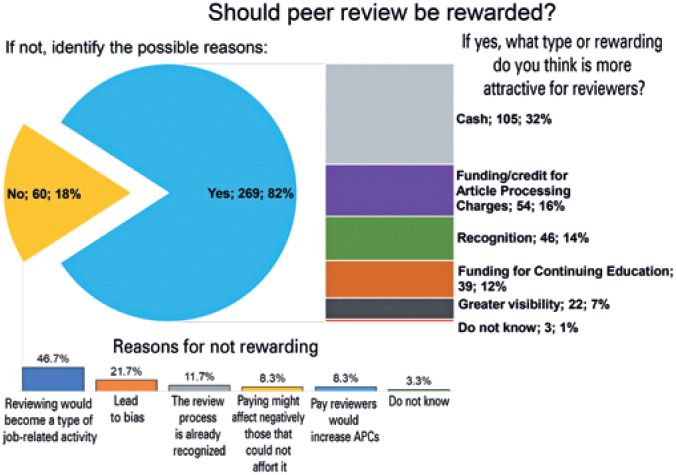
Types of preferable rewards and reasons for not rewarding

Regarding rewarding, the majority of respondents (269) favored rewarding reviewers, and the main choices of compensations were money (105) and/or credits for future publications (54). However, some respondents (60) expressed concerns that payment *would shift reviewing to a type of job-related activity rather than a service for the scientific community* (46.7%) and/or that "rewarding may lead to some type of bias" (21.7%) (Figure 2).

## Is there a solution?

The factors *time*, *subject of the paper*, and *prestige of the journal* seem to be most important on accepting or not to peer review a research article. Editors-in-Chief should define strategies to contribute with reviewers to overcome challenges, and, consequently, increase the number of acceptance and reduce rejections on reviewing a paper.

The advent of increased popularity in the adoption of Artificial Intelligence systems for a number of activities might be beneficial for peer review.^(7)^ These systems, like ChatGPT, can support reviewers in quality-improvement and gatekeeping about the publishability of a paper. Other activities that these systems are currently able to do may include providing feedback on specific parts of the manuscript, matching reviewers with papers based on keywords by combining reviewer's scientific output with the paper's subject matter, and automating invitation processes. These support may result in alleviating burden for editors and reviewers.^(7)^

The preference for monetary and credit compensation among reviewers is not unique to einstein (São Paulo)'s survey. Various suggestions have been advocated, such as the ‘US$450 Movement’ proposed by James Heathers during the Research to Readers conference in 2020.^(8)^ Basically, this movement suggests that US$450 would be a reasonable amount to be paid to reviewers. However, this amount would be impracticable for small publishers and journals, especially those in periphery and semi-periphery countries, like Brazil. Nevertheless, there is always room for negotiation, and publishers may find a balance between budget constraints and reviewer satisfaction.

It is paramount to carefully consider the potential impacts on the publishing activity that the monetary compensation may have, including the possibility of increased article processing charges as a way to cover the cost related with paying reviewers. This may result in a serious funding problem, which in science, in a number of areas, is already constrained.

Around the world, every professional activity, which requires sophisticated knowledge, has a monetary compensation. For this reason, payment, within reasonable limits, should be adopted and encouraged, as surveys, like the one conducted by einstein (São Paulo), have shown. Multinational publishing giants such as Springer Nature, Taylor & Francis, Wiley, and Elsevier Inc, of which the latter, has an annual income surpassing £6 billion,^(9)^ could take the lead in initiating this conversation. Ultimately, ensuring fair compensation to a complex and time-consuming activity, such as peer review, the final product of which is expected to be of high quality, seems to be crucial for sustaining the integrity and quality of academic publishing. Undoubtedly, this topic deserves to be further discussed by the scientific community and global publishing market.
